# Ameloblastoma of the Mandible in a 16-Year-Old Female—Case Report

**DOI:** 10.3390/medicina60010066

**Published:** 2023-12-29

**Authors:** Horatiu Urechescu, Ancuta Banu, Flavia Baderca, Raluca Maria Closca, Maria-Bianca Ilas-Tat, Florin Urtila, Marius Pricop

**Affiliations:** 1 Department of Oral and Maxillo-Facial Surgery, Faculty of Dental Medicine, “Victor Babes” University of Medicine and Pharmacy Timisoara, Eftimie Murgu Square No. 2, 300041 Timisoara, Romania; urechescu.horatiu@umft.ro (H.U.); urtila.florin@umft.ro (F.U.); pricop.marius@umft.ro (M.P.); 2 Department of Microscopic Morphology, “Victor Babes” University of Medicine and Pharmacy Timisoara, Eftimie Murgu Square No. 2, 300041 Timisoara, Romania; baderca.flavia@umft.ro (F.B.); raluca.moaca@umft.ro (R.M.C.); 3 Angiogenesis Research Center, “Victor Babes” University of Medicine and Pharmacy Timisoara, Eftimie Murgu Square No. 2, 300041 Timisoara, Romania; 4 Service of Pathology, Emergency City Hospital, 300254 Timisoara, Romania; drtatbianca0@gmail.com

**Keywords:** ameloblastoma, mandible, COVID-19

## Abstract

Ameloblastoma is a benign epithelial tumor that has aggressive, destructive and unlimited growth potential, having the capacity for recurrence and malignant transformation. Regarding the symptoms and clinical signs, the presentation of ameloblastoma is poor. In children and young people, ameloblastoma can be difficult to diagnose, because it mimics other benign lesions. Its diagnosis requires a combination of imaging data, histopathological analysis and molecular tests. The methods of treatment consist of radical surgery (segmental resection) and conservative treatments (enucleation with bone curettage). The particularity of the presented case is represented by the late request for medical consultation, a direct consequence of the measures implemented to prevent and control the spread of COVID-19.

## 1. Introduction

Ameloblastoma is a tumor whose terminology has been modified over time. Establishing the nomenclature of ameloblastoma based on clinical and histological characteristics and biological behavior is still a topic of interest among oral pathologists and molecular biologists [1]. Ameloblastoma arises from remnants of the odontogenic epithelium, more specifically the remnants of the dental lamina. Remnants situated outside the bone in the soft tissues of the gingiva or alveolar mucosa may give rise to peripheral ameloblastoma. Other possible sources of origin include the gingival surface epithelium and lining of odontogenic cysts [2]. Genetic studies have made a significant contribution to updating the types of ameloblastoma. The pathogenesis of ameloblastoma has been found to be related to the deregulation of the SHH, WNT/β-catenin and MAPK signaling pathways, and the detection of the BRAF V600E mutation has been found to be associated with more aggressive clinical forms [3].

There are data in the literature that indicate LRP5, SLC6A3 and SOX10 are potentially important genes whose presence is related to cell proliferation and invasion of ameloblastomas, and a possible inhibitory treatment is indicated subject to clarification of the molecular pathways of these genes in relation to ameloblastoma tumorigenesis [4].

Ameloblastoma classification has been narrowed to conventional ameloblastoma, unicystic and extraosseous/peripheral types. The solid/multicystic type was eliminated because most conventional ameloblastomas show cystic degeneration with no biological differences. The desmoplastic type was left under the histopathological subtype (follicular, plexiform, acanthomatous, granular cell, basaloid and desmoplastic) rather than as a separate entity. Ameloblastic carcinoma is now classified under ameloblastic carcinomas due to the morphologic continuum and similar behavior between these entities. Metastasizing ameloblastoma has been moved to benign ameloblastoma subtypes from malignant odontogenic tumors (the main reason behind this change is attributed to the fact that primary and metastatic ameloblastomas are histopathologically identical to benign ameloblastoma). Odontoameloblastoma, which was used in the 2005 WHO classification, is no longer used because the ameloblastic areas in the odontoma do not justify a separate entity [3].

A quick search of the literature highlights 67 case reports related to mandibular ameloblastoma since 2017, when the classification of ameloblastoma was last updated. This report highlights the fact that the patient delayed presentation to the doctor due to the measures instituted at the national level during the COVID-19 pandemic. Although pain and swelling are symptoms that normally cause patients to request an urgent consultation, in this case, the patient decided to postpone the medical consultation until the restrictions related to COVID-19 were lifted. During this time, the injury evolved, increasing asymmetry. Another particularity of this case is the conservative treatment used, preserving the continuity of the mandible.

## 2. Case Report

We present the case of a 16-year-old girl who presented to the Maxillofacial Surgery Department complaining of swelling of the right perimandibular region for the last two years.

The particularity of this case is represented by the late request for medical consultation, a direct consequence of the measures implemented to prevent and control the spread of COVID-19, which, through restrictions implemented at the state level, complicated or even blocked patients’ ability to acquire medical assistance other than that related to COVID-19. Even after the elimination of restrictions, through the changes produced in the collective mind, the means of limiting access to medical services persisted.

**Figure 1:**
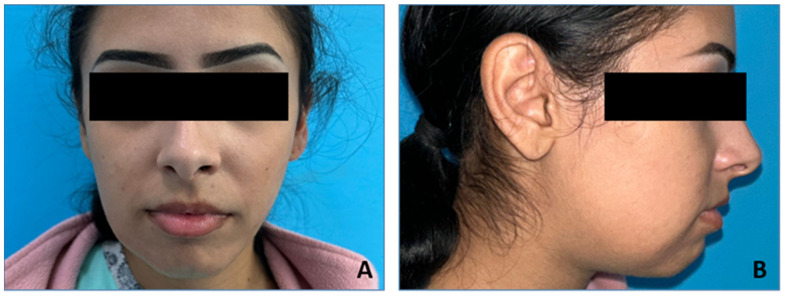
The facial examination. (**A**) Frontal view; (**B**) lateral right view.

**Figure 2:**
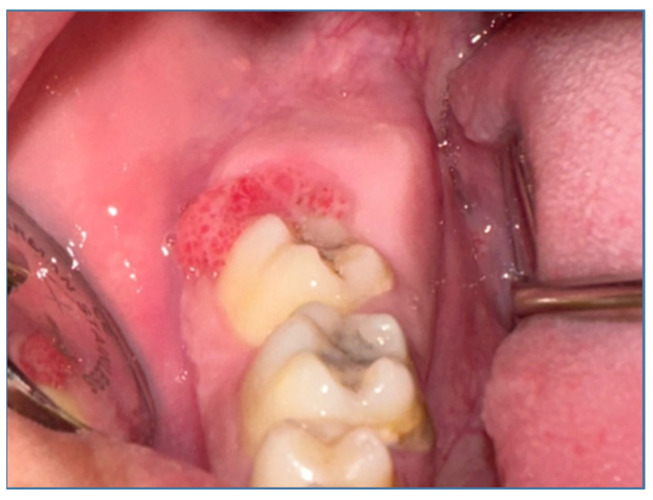
Intraoral examination.

The facial examination (Figure 1) showed an asymmetry caused by a large diffuse swelling located in the right mandibular region, hard in consistency and painless. The skin overlying the swelling was stretched and was of normal color. The swelling started spontaneously and gradually increased to its current size. There was no history of trauma or dental pain. The patient was experiencing pain while chewing and did not have altered sensation over the right lip region. The intraoral examination (Figure 2) revealed a swelling in the right lower posterior buccal vestibule extending from the second premolar to the retromolar region. The overlaying mucosa presented a superficial ulceration distal from the second molar. The laboratory tests, consisting of a complete blood count and biochemical and coagulation profiles, were all within normal limits. The only test that showed increased values was ESR = 24 mm/h (0–10 mm/h). Also, the procalcitonin (PCT) value was slightly increased at 0.41 (0.17–0.35/fL). This showed a degree of superinfection. The interdisciplinary cardiological consultation, including the EKG, did not show any changes. The chest X-ray showed no changes. No cervical ultrasound was performed, the cervical ganglion system being clinically unchanged.

**Figure 3:**
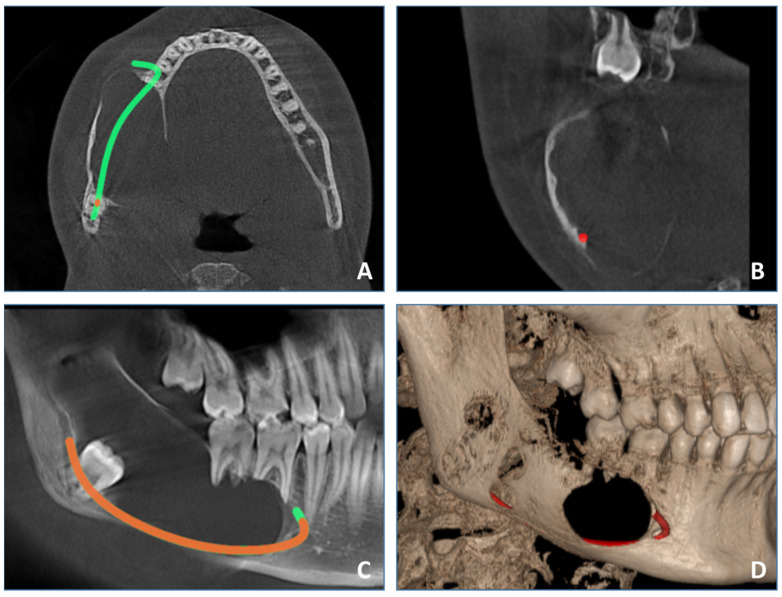
CBCT examination. (**A**)—axial view, (**B**)—coronal view, (**C**)—sagittal view, (**D**)—3D reconstruction.

The patient underwent cone beam computed tomography (CBCT), which provides a detailed 3D analysis of the dentition and cortical and medullary bone free of superimpositions compared with classic radiographs like OPG. Compared with other cross-sectional imaging modalities like spiral computed tomography or MRI, it is easily available, relatively inexpensive and generally has a lower radiation dose. CBCT examination (Figure 3) revealed a well-defined expansive radiolucency involving the right mandible, measuring approximately 64 mm/25 mm/26 mm centimeters (sagittal/transversal/vertical). The lesion spans in the sagittal plane from the periapical region of the first right mandibular molar, involving the body, angle and ramus, to the sigmoid notch, vertically from the crest of the mandibular ridge to the lower border of the mandible. Bucco-lingual expansion was noted with extensive thinning of cortical plates and perforation of cortical plates. Displacement of impacted lower right third molar inferiorly into the lower border of the mandibular angle was observed. Resorption of the roots of the first and second right mandibular molars was noted. The inferior alveolar nerve canal was displaced inferiorly.

The clinical and imagistic findings could have been suggestive of ameloblastoma, but an odontogenic cyst could not be excluded. The main differential diagnosis that was considered was an odontogenic keratocyst. In this case, the risk of pathological fracture of the mandible is increased. It was decided that the patient should undergo emergency surgery, which is why no preoperative biopsy was performed. The lesion was surgically enucleated with curettage and extraction of the first and second right mandibular molars and third impacted right mandibular molar under general anesthesia. The informed consent for surgery was obtained from the patient’s mother.

The harvested specimens were fixed in 4% *v*/*v* buffered formaldehyde, sent to pathology services and processed with the usual histological technique. Four-micrometer-thick sections were cut using a semi-automated Leica RM2235 rotary microtome, displayed on SuperFrost™ microscope (Fisherbrand, NH, USA) slides and stained with hematoxylin and eosin.

**Figure 4:**
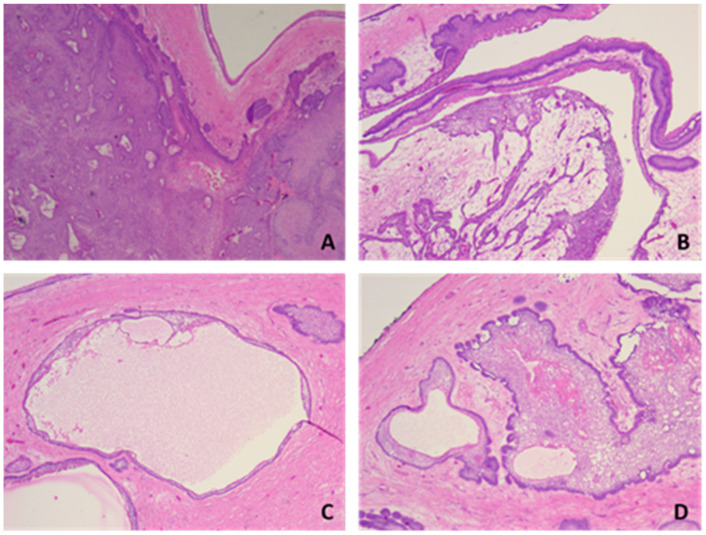
Microscopic aspects of ameloblastoma and different growth patterns: (**A**) solid, (**B**) plexiform, (**C**) macrocystic and (**D**) acanthomatous. HE staining, ob. 10×.

**Figure 5:**
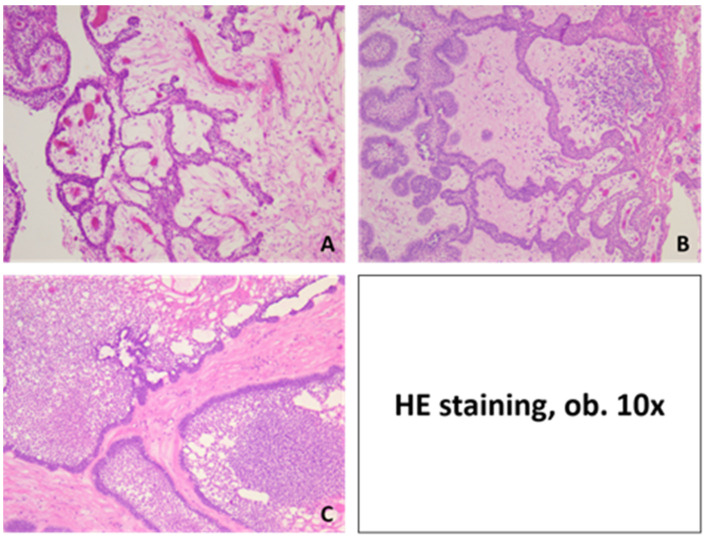
Histopathological aspects of ameloblastoma: (**A**,**B**) plexiform pattern, with anastomosing cords and trabeculae of odontogenic epithelial cells scattered in connective loose tissue with inflammatory cells; (**C**) acanthomatous pattern with squamous metaplasia and unicellular keratinization. HE staining, ob. 10×.

**Figure 6:**
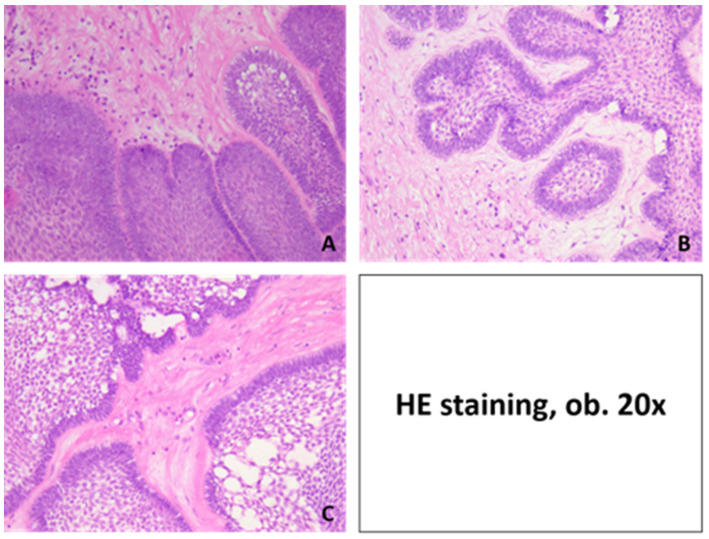
Histopathological aspects of ameloblastoma: (**A**) acanthomatous pattern with squamous metaplasia and unicellular keratinization; (**B**) follicular growth pattern, centrally stellate reticulum-like; (**C**) acanthomatous pattern with squamous metaplasia, stellate cells and microcysts. HE staining, ob. 20×.

**Figure 7:**
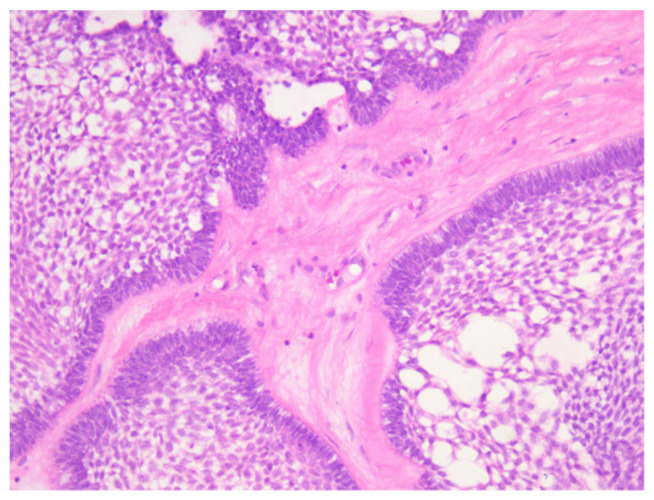
Microscopic aspects of ameloblastoma–acanthomatous pattern with squamous metaplasia, stellate cells and microcysts. HE staining, ob. 40×.

Histopathological examination showed tumor proliferation with a follicular, plexiform and solid (acanthomatous) pattern, with cyst formation. In a follicular pattern, islands of odontogenic epithelium were interspersed within a stoma of mature collagenous connective tissue. The islands had columnar cells at the periphery with reverse polarity and nuclei oriented away from the basal membrane, and the central portion of the islands presented loosely arranged epithelium cells that resemble the stellate reticulum of the developing enamel organ. The plexiform pattern showed an odontogenic epithelium arranged in long cords and stands that surrounded central areas of the supporting stroma. In addition to the interconnecting epithelium stands and cords, islands and sheets of tumors cells were observed. Also, the tumor showed areas of cystic degeneration, with the formation of micro- or macrocysts. Some islands of tumor cells demonstrated central areas of squamous differentiation, with the formation of keratotic pearls or individual keratinizations (Figure 4, Figure 5, Figure 6 and Figure 7). There were no signs of malignant transformation and no immunohistochemical reactions.

Short-term follow-up 4 months after surgery revealed a healed surgical site with no complaint of numbness of the right lower lip. Follow-up clinical and radiographic examination is essential as ameloblastoma has a high recurrence rate. Depending on the follow-up findings, a wide-margin excision and reconstruction can be performed. Also, prosthetic rehabilitation is further planned for the patient to correct the surgical defect and missing teeth.

## 3. Discussion

Ameloblastoma is a benign epithelial tumor that constitutes about 14% of all jaw tumors and cysts. It has aggressive, destructive and unlimited growth potential, having the capacity for recurrence, malignant transformation and metastasis (in approximately 1% of cases). There is no differentiation according to sex. The global incidence of ameloblastoma is 0.5 cases/million people, with 10–15% of cases occurring in the pediatric population, reaching up to 25% in Africa and Asia [4].

Regarding intraosseous gnathic ameloblastoma (conventional and unicystic), ~80% of cases occur in the mandible and ~20% cases occur in the maxilla [5]. Very rarely is it reported in other head and neck sites like the sinonasal tract [6], middle ear [7], temporal bone [8] and infratemporal fossa [9].

The maximum incidence depending on age varies as follows: conventional type ameloblastoma, between 40 and 50 years; unicystic type ameloblastoma, between 20 and 30 years; and extraosal/peripheral type ameloblastoma, between 50 and 70 years [10].

Regarding the symptoms and clinical signs, the presentation of ameloblastoma is poor. In some cases, a radiological change is occasionally detected on radiographs taken for other reasons [11]. Painless swelling, with slow regional bone growth, is the most common presenting symptom of ameloblastoma. Invasion of soft tissues, mobility of adjacent teeth and dental malocclusion are other clinical signs. Pain is an unusual symptom that can occur as a result of hemorrhage inside or adjacent to the tumor, or as a result of the invasion of some nerve structures [12].

In children and young people, ameloblastoma can be difficult to diagnose, because it mimics other benign lesions. Its diagnosis requires a combination of imaging data, histopathological analysis and molecular tests. One example is the second most common type of ameloblastoma, unicystic ameloblastoma. Located predilected in the posterior mandibular area, it is clinically and radiologically similar to a dentigerous cyst due to its association with impacted teeth [13]. One study has associated ameloblastoma with an impacted tooth in 70% to 83% of cases [14].

The prognosis for ameloblastoma varies depending on age, type, location and size of the formation, in direct relation to the degree of bone involvement, damage to adjacent structures and type of surgical intervention (radical or conservative). All these influence the recurrence rate of ameloblastoma, which is approximately 10% and can increase to values between 55% and 90%, which complicates the patient’s clinical condition in the long term [15,16]. The granular and follicular histological subtypes exhibit a higher recurrence rate. Inadequately short follow-up periods may mislead physicians to falsely believe the patient is cured, potentially leading to the oversight of metastatic ameloblastoma [17,18].

Radiologically, unicystic ameloblastoma has the following characteristics: unilocular radiolucency, well-defined, corticated border, often associated with an impacted tooth, specifically the mandibular third molar, possible root resorption and cortical perforation in 33% of cases.

According to some studies prior to 2020, ameloblastomas had an average size of approximately 4 cm at presentation [19]. A meta-analysis shows an average ameloblastoma-specific growth rate of 87.84% per year [20].

Regarding the presented case, its particularity is represented by the late request for medical consultation, a direct consequence of the measures implemented to prevent and control the spread of COVID-19. Coronavirus disease 2019 (COVID-19) was accompanied by a series of measures that put pressure on health systems around the world. The available medical assistance capacity was affected by the conversion of medical units into COVID-19 care units [21], the deployment of doctors to care for patients with COVID-19, the limitation of the number of hospitalizations by increasing the hospitalization time until obtaining the result of the RT-PCR test and the decrease the degree of occupation of medical departments by isolating patients. Other notable changes that affected patients included the postponement of routine care, the use of telemedicine as a substitute for clinical consultation with a negative impact on the quality of care, the restriction of the activity of dental offices, triage activities [22]. Also, the fear of viral contagion produced a reluctance of patients to request medical care [23]. It is reported in various medical studies that these changes have led to diagnostic delays and poorer therapeutic results in a number of conditions [24,25,26].

## 4. Conclusions

Ameloblastoma is a benign epithelial tumor that has an aggressive, destructive, unlimited growth potential, having the capacity for recurrence and malignant transformation. The diagnosis requires a combination of imaging data and histopathological analysis to be confirmed. The methods of treatment consisted of radical surgery (segmental resection) and conservative treatments (enucleation with bone curettage). The particularity of the presented case is represented by the late request for medical consultation, a direct consequence of the measures implemented to prevent and control the spread of COVID-19, which led to diagnostic delays and poorer therapeutic results. Periodic clinical and radiological follow-up are mandatory due to the high recurrence rate of ameloblastoma.

## Data Availability

The data generated in this study may be requested from the corresponding author.
